# Prospective acceleration of diffusion tensor imaging with compressed sensing using adaptive dictionaries

**DOI:** 10.1002/mrm.25876

**Published:** 2015-08-24

**Authors:** Darryl McClymont, Irvin Teh, Hannah J. Whittington, Vicente Grau, Jürgen E. Schneider

**Affiliations:** ^1^ Division of Cardiovascular MedicineRadcliffe Department of MedicineUniversity of OxfordOxfordUnited Kingdom; ^2^ Department of Engineering ScienceUniversity of OxfordOxfordUnited Kingdom

**Keywords:** diffusion MRI, diffusion tensor imaging, prospective undersampling, compressed sensing, adaptive dictionaries, heart structure

## Abstract

**Purpose:**

Diffusion MRI requires acquisition of multiple diffusion‐weighted images, resulting in long scan times. Here, we investigate combining compressed sensing and a fast imaging sequence to dramatically reduce acquisition times in cardiac diffusion MRI.

**Methods:**

Fully sampled and prospectively undersampled diffusion tensor imaging data were acquired in five rat hearts at acceleration factors of between two and six using a fast spin echo (FSE) sequence. Images were reconstructed using a compressed sensing framework, enforcing sparsity by means of decomposition by adaptive dictionaries. A tensor was fit to the reconstructed images and fiber tractography was performed.

**Results:**

Acceleration factors of up to six were achieved, with a modest increase in root mean square error of mean apparent diffusion coefficient (ADC), fractional anisotropy (FA), and helix angle. At an acceleration factor of six, mean values of ADC and FA were within 2.5% and 5% of the ground truth, respectively. Marginal differences were observed in the fiber tracts.

**Conclusion:**

We developed a new k‐space sampling strategy for acquiring prospectively undersampled diffusion‐weighted data, and validated a novel compressed sensing reconstruction algorithm based on adaptive dictionaries. The k‐space undersampling and FSE acquisition each reduced acquisition times by up to 6× and 8×, respectively, as compared to fully sampled spin echo imaging. Magn Reson Med 76:248–258, 2016. © 2015 Wiley Periodicals, Inc.

## INTRODUCTION

Diffusion MRI (dMRI) is a noninvasive technique that measures the displacement of water molecules as a marker of cell orientation and integrity. In the myocardium, fiber and sheet orientation have been shown to coincide with the eigenvectors of the diffusion tensor 1, 2. In diffusion tensor imaging (DTI), diffusion‐weighted (DW) measurements are performed in a minimum of six directions plus one nondiffusion weighted scan, and the data are fitted with a monoexponential tensor model 3. As a result, scan times can be long, limiting the clinical application of DTI. This has led to the development of several methods for accelerating cardiac dMRI including reduced encoding sequences 4, parallel imaging 5, and simultaneous multislice imaging 6.

In recent years, compressed sensing (CS) has emerged as a popular method for reconstructing undersampled MRI data 7. The requirements for CS reconstruction of undersampled MRI data are that the images are sparse in a specific transform domain, and that the reconstruction artefacts from the transform are incoherent with those from undersampling 7.

The problem of how best to sparsify dMRI signals has been widely discussed in recent years. Several studies use wavelet transforms for sparsifying the diffusion propagator 8, 9 or signal in image‐space 10. Spherical ridgelets, based on wavelet theory, also offer a means to sparsely represent diffusion signals 11. Recently, Mani et al 12 proposed a compressed sensing reconstruction algorithm based on a multitensor model 13, where the diffusion signal at each voxel was modelled using contributions from a dictionary consisting of one isotropic component and several anisotropic components. However, such dictionaries were intended for application in white matter in the brain, and their suitability for the heart is unclear given the lower anisotropy of cardiac tissue 14. Adaptive dictionaries 15, 16 provide a method of sparsely representing diffusion signals without making assumptions about the underlying diffusion processes.

In dMRI, undersampling may be performed in k‐space 8, 10, 12, 17, 18, 19, or in q‐space 13, 15, 16, 20, 21, 22, 23. Q‐space undersampling is generally applied when the number of diffusion‐encoding directions is high, such as in high angular resolution diffusion imaging or diffusion spectrum imaging. When the number of diffusion‐encoding directions is low, k‐space undersampling is more commonly performed. Furthermore, a different k‐space sampling scheme may be used to acquire each image volume so as to decrease coherence in data sampling 8, 10, 12, 18.

To the best of the authors' knowledge, previous published work accelerating DTI with CS have been limited to retrospectively undersampled data. In this work, we present and evaluate a complete system for prospectively accelerated acquisition and reconstruction of three‐dimensional (3D) DTI data, with the aim of reducing total acquisition time. The acquisition combines variable density k‐space undersampling with a fast spin echo sequence. The reconstruction algorithm exploits sparsity by means of adaptive dictionaries 24, 25, and incorporates T_2_‐weighting correction. The T_2_‐weighting correction supports fast echo‐train imaging methods, and leads to greater flexibility in k‐space trajectory designs. We compared retrospectively and prospectively undersampled ex vivo cardiac data, and validated the reconstructions against fully sampled data acquired during the same session. A preliminary version of this work was presented in McClymont et al 26.

## METHODS

### MR Setup and Acquisition

MRI was performed using a 9.4 Tesla (T) preclinical scanner (Agilent Technologies, Santa Clara, CA), a shielded gradient system (1 T/m) and a quadrature‐driven transmit/receive birdcage coil of 20 mm inner diameter (Rapid Biomedical, Rimpar, Germany). Fully sampled 3D fast spin echo (FSE) DTI data were acquired with the following parameters: TR/TE = 250/9.3 ms, echo spacing = 4.9 ms, echo train length = 8, resolution = 100 μm isotropic, acquisition matrix ( $k_{x}\;\times \;k_{y}\;\times \;k_{z})$ = 200 × 160 ×160, field‐of‐view = 20 × 16 ×16 mm, number of non‐DW images = 8, number of DW directions = 61, diffusion duration (δ) = 2 ms, diffusion time (Δ) = 5.5 ms, b‐value = 1000 s/mm^2^, acquisition time = 15 h 20 m. The receiver gain was optimized for DW and non‐DW scans. Noise data were acquired using an identical sequence, without radiofrequency (RF) pulses and with TR = 67 ms.

Prospectively undersampled data were acquired at 2×, 3×, 4×, 5×, and 6× acceleration factors, with a different randomized undersampling mask for each image volume. The parameters used were identical to the fully sampled data, with the following differences: number of non‐DW images = 4, number of DW directions = 30. The diffusion scheme used here corresponded to the first 30 of 61 DW directions of the fully sampled data 27. The acquisition times were 3 h 46 min, 2 h 31 min, 1 h 53 min, 1 h 30 min, and 1 h 15 min, respectively. In addition, 3D multiecho spin echo data were acquired for T_2_ mapping. The following parameters were used: TR/TE_1_ = 250/4.7 ms, echo spacing = 4.7 ms, echo train length = 16, resolution = 100 μm isotropic.

### k‐Space Sampling

**Figure 1:**
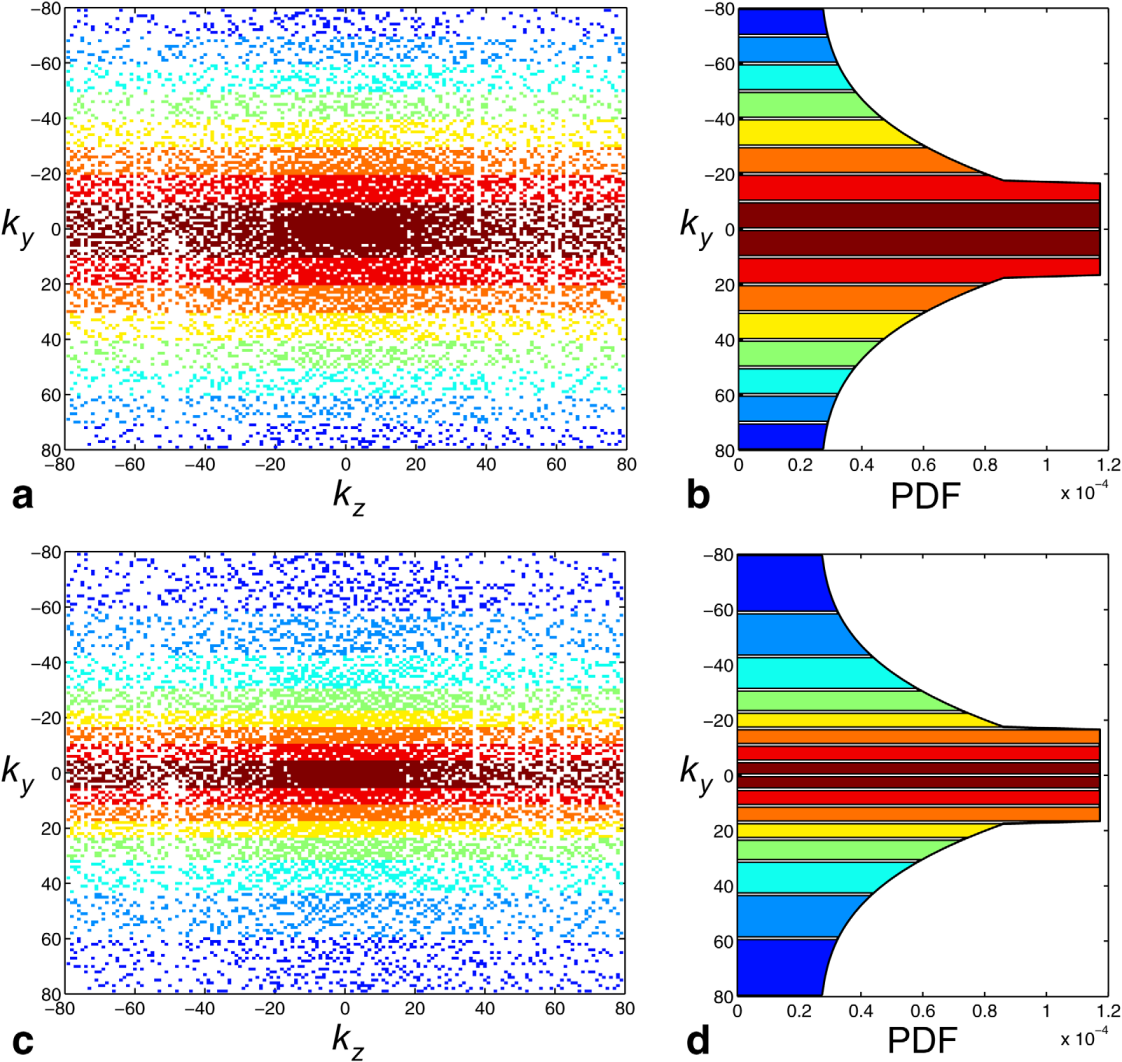
Prospective and retrospective undersampling (US) schemes. **a**: An example sampling mask of the retrospective 2× undersampling scheme, color‐coded by echo number (red corresponds to the first echo, blue to the eighth). The read‐out, $k_{x}$, is fully sampled. Un‐sampled locations are coloured white. **b**: The sampling probability density function (PDF) along $k_{z}\;=\;0$, and the corresponding bands of uniform width in k_y_. **c,d**: The sampling mask and sampling PDF for prospective undersampling, and the corresponding bands whose areas under the curve are approximately equal.

In an FSE acquisition, the number of echoes per excitation is fixed. In the fully sampled datasets, the two phase‐encoding directions $k_{y}$ and $k_{z}$ were traversed in a standard center‐out interleaved and linear manner, respectively. This resulted in a stepped filter of equal width bands across $k_{y}$ modulated by T_2_ relaxation. To implement the prospectively undersampled DTI sequence, a sampling scheme was generated that divides $k_{y}$ into bands of variable widths, whilst maintaining a center‐out phase encoding scheme to mitigate discontinuities in T_2_‐weighting. Figure 1 illustrates the sampling schemes for both retrospectively and prospectively undersampled data. First, a probability density function (PDF) was generated in $k_{y},k_{z}$ for each acceleration factor, f. Following 10, this PDF was constructed using a polynomial of order f+1, with the central 15% of k‐space fully sampled. In the prospectively undersampled case, bands in $k_{y}$ were defined such that the integral of the PDF in each band was approximately equal at $k_{z}\;=\;0$. Although the same PDF was used for each imaging volume, the sampling schemes were generated independently to decrease coherence. Samples were randomly allocated based on the PDF, first in $k_{z}$ and then in $k_{y}$, independently for each half of k‐space ( $k_{y}>0$ and $k_{y}\leq 0$). Each read‐out ( $k_{x}$) was fully sampled. The retrospectively undersampled k‐space data were sampled in the same locations as the prospectively undersampled data.

### Notation

Matrices and arrays are denoted using upper case bold letters; vectors are denoted using lower case bold. Images are represented interchangeably as arrays, as in $I\subseteq {\mathbb{Z}}^{X\times Y\times Z\times N}$, or as matrices, as in $I\subseteq {\mathbb{Z}}^{N\times V}$ where V=X×Y×Z is the total number of voxels. X, Y, and Z refer to the dimensions of the data in the read‐out and two phase encoding directions, respectively. N refers to the number of image volumes including DW and non‐DW data. Elements of arrays are denoted using subscripts, such as $I_{x,y,z,n}$. Column v of matrix I is denoted $i_{v}$.

### Image Reconstruction

**Figure 2:**
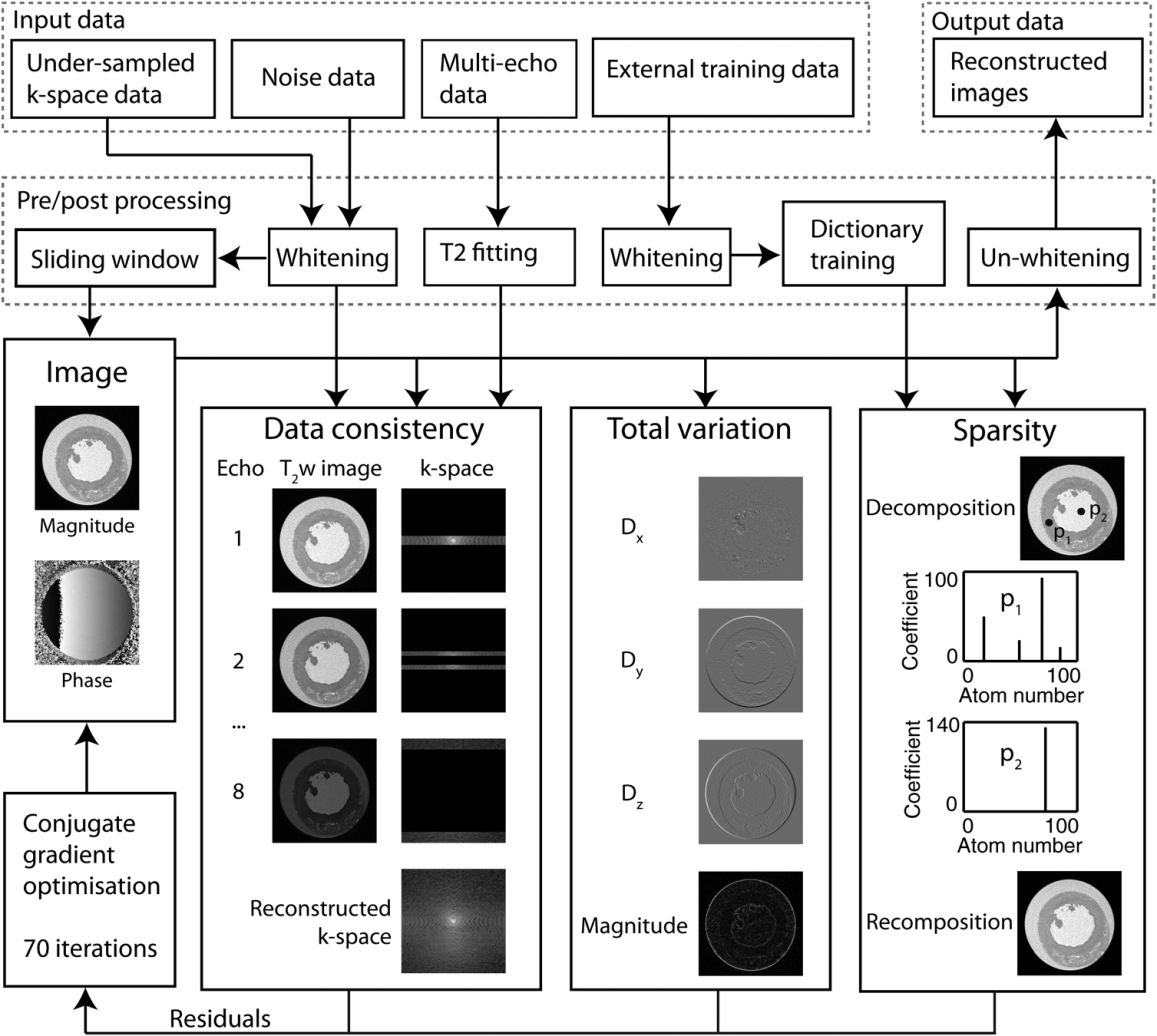
Flowchart describing the compressed sensing reconstruction. Images are reconstructed by simultaneously minimizing terms corresponding to data consistency, sparsity, and total variation. In the data consistency module, the estimated k‐space consists of the sum of contributions from each echo. In the total variation module, the sum of finite differences in the magnitude image is minimized. In the sparsity module, two example points are displayed. Point p_1_ corresponds to a voxel in the myocardium, and requires four dictionary atoms to be adequately represented. Point p_2_ is in the buffer, and can be represented by a single dictionary atom.

Images were reconstructed using the compressed sensing framework shown in Figure 2. The input k‐space data to the reconstruction algorithm were initialized using a “sliding window” approach 8, 28, in which unsampled locations are filled with those of the nearest acquired diffusion direction. This was performed independently for the non‐DW and DW data.

Although noise in magnitude MR images follows a Rician distribution, given a sufficiently high signal‐to‐noise ratio (SNR) it can be approximated as Gaussian 29. However, the different receiver gain settings introduce heteroscedasticity between the non‐DW and DW data. Therefore, data whitening must first be performed so that errors can be assumed to follow a Gaussian distribution, and can, therefore, be minimized using the computationally efficient ℓ_2_‐norm. The noise level at each receiver gain was computed as the standard deviation of the real component of the noise data in k‐space. Before reconstruction, the non‐DW and DW images were whitened by dividing the data in k‐space by the appropriate noise level. Following reconstruction, the images were unwhitened by multiplying by the respective noise level.

The complex diffusion weighted images, $I\subseteq {\mathbb{Z}}^{X\times Y\times Z\times N}$, were reconstructed by solving the following optimization problem:

(1) $$I^{*}=\underset{I}{\mathrm{argmin}}\;{\left\|F\left(I\right)-Y\right\|}_{2}^{2}+\lambda_{1}{\left\|\left|I\right|\right\|}_{\mathrm{TV}}+\lambda_{2}{\left\|\phi \left(\left|I\right|\right)\right\|}_{1}.$$

The first term in Eq. [1] refers to data consistency, where Y denotes the whitened k‐space data, and $F\left(I\right)$ is the k‐space of the reconstructed image, as follows:

(2) $$F_{k_{x},k_{y},k_{z},n}\left(I\right)=\sum_{x,y,z}^{}I_{x,y,z,n}\;e^{-i2\pi \left(\frac{k_{x}x}{X}+\frac{k_{y}y}{Y}+\frac{k_{z}z}{Z}\right)}\;e^{-\frac{TE\left(k_{y}\right)-TE(1)}{{T2}_{x,y,z}}}.$$

This definition differs from the discrete Fourier transform in that it incorporates the echo time, $TE\left(k_{y}\right)$, at each phase encoding location, and the T_2_ at each voxel, ${T2}_{x,y,z}$. This term mitigates the ringing and blurring artefacts due to discontinuous T_2_‐weighting in k‐space 30, 31. T_2_ maps were obtained by fitting the multiecho spin echo data with a monoexponential relaxation curve using weighted linear regression. The $TE\left(1\right)$ term allows the approximation of the T_2_ relaxation that would have occurred, had all of the data been acquired at the TE of the first echo.

The second term in Eq. [1] controls image smoothness. The total variation (TV) was computed from the magnitude of the diffusion‐weighted images $\left|I\right|$ as follows:

(3) $${\left\|\left|I\right|\right\|}_{\mathrm{TV}}=\sum_{x,y,z,n}^{}\left|\left|I_{x+1,y,z,n}\right|-\left|I_{x,y,z,n}\right|\right|+\left|\left|I_{x,y+1,z,n}\right|-\left|I_{x,y,z,n}\right|\right|+\left|\left|I_{x,y,z+1,n}\right|-\left|I_{x,y,z,n}\right|\right|.$$

Finally, the third term in Eq. [1] controls the sparsity of the image under the transformation ϕ. Here, ${\left\|\phi \left(\left|I\right|\right)\right\|}_{1}$ refers to the ℓ_1_‐norm of the linear decomposition by a dictionary, as described in the following section. Tuning parameters $\lambda_{1}$ and $\lambda_{2}$ control the relative weights of the total variation and sparsity terms.

### Sparse Representation

Let $x\subseteq {\mathbb{Z}}_{}^{N\times 1}$ denote the magnitude of the diffusion‐weighted signal at a particular voxel. Following Gramfort et al 16, x was modeled as follows:

(4) x=Da+ε

where $D\subseteq {\mathbb{Z}}_{}^{N\times D}$ refers to the dictionary, $a\in {\mathbb{Z}}_{}^{D\times 1}$ are the coefficients, and $\varepsilon \subseteq {\mathbb{Z}}_{}^{N\times 1}$ is a noise term. The coefficient vector a should be sparse (i.e., use as few columns of the dictionary as possible), while minimizing the magnitude of the residuals ε. Given that x is strictly nonnegative, it follows that D and a are also nonnegative 16.

Given a set of whitened training data $X=[x_{1},\ldots ,x_{V_{\mathrm{tr}}}]\subseteq {\mathbb{Z}}_{}^{N\times V_{\mathrm{tr}}}$, the dictionary was trained as follows 25:

(5) $$D^{*},A^{*}=\underset{D\geq 0,A\geq 0}{\mathrm{argmin}}\;\frac{1}{V_{\mathrm{tr}}}\sum_{v}{\left\|a_{v}\right\|}_{0}\;s.t.\;{\left\|x_{v}^{}-Da_{v}\right\|}_{2}^{2}\leq N$$

where $A=\left[a_{1},\ldots ,a_{V_{\mathrm{tr}}}\right]\subseteq {\mathbb{Z}}_{}^{{D\times V}_{\mathrm{tr}}}$ contains the sparse coefficients. As the data was whitened such that the noise has a variance of 1, the residual sum of squares should be less than or equal to N. To prevent D from having arbitrarily large values, its columns $d_{1},\;\ldots ,\;d_{D}$ are constrained to each have an ℓ_2_‐norm of less than or equal to one.

During reconstruction, the magnitude image $\left|I\right|\subseteq {\mathbb{Z}}_{}^{N\times V}$ in Eq. [1] was ℓ_0_‐“norm” decomposed using iteratively re‐weighted ℓ_1_‐norm decomposition 32, 33. The decomposition was performed using the trained dictionary as follows:

(6) $$A^{*}=\underset{A\geq 0}{\mathrm{argmin}}\;{\left\|WA\right\|}_{1}\;s.t.\;{\left\|\left|I\right|-DA\right\|}_{2}^{2}\leq \lambda$$

where $W=\frac{1}{A+\varepsilon }$. The stability parameter, ε, was 0.01. Thus, ${\left\|WA\right\|}_{1}\approx {\left\|A\right\|}_{0}$. The error term, λ, was N in the first iteration of the reconstruction algorithm. In subsequent iterations, λ was equal to $\frac{N}{V}{\sum_{v}\left\|\left|I_{v}\right|-DA_{v}\right\|}_{2}^{2}$, using A from the previous iteration.

The sparsity term, ${\left\|\phi \left(\left|I\right|\right)\right\|}_{1}$, in Eq. [1] is defined as $\sum \left({\left\|A\right\|}_{1}+{\left\|\left|I\right|-DA\right\|}_{1}\right)$. This is equivalent to representing residuals using additional columns of the dictionary, $d_{D+1}={\left[1,\;0,\;\ldots ,\;0\right]}^{\text{T}}$, $d_{D+2}={\left[0,\;1,\ldots ,\;0\right]}^{\text{T}}$, …, $d_{D+N}={\left[0,\;0,\ldots ,\;1\right]}^{\text{T}}$, thus maximizing their ℓ_1_‐norm.

### Iterative Reconstruction

Nonlinear conjugate gradient descent was used to perform the reconstruction. Seventy iterations were typically sufficient for convergence. The tuning parameters $\lambda_{1}$ and $\lambda_{2}$ were 1 and 10 in the 2D phantom reconstructions, and $10^{-4}$ and 0.1 in the 3D ex vivo data reconstructions, respectively. The number of dictionary columns, D, was 100. These parameters were empirically tuned using additional data, not included in this study. Dictionary learning and sparse decomposition was performed using the SPAMS toolbox of Mairal et al 25, using the weighted LASSO algorithm (34) for sparse decomposition. The ℓ_0_‐norm decomposition required only two iterations of weighted ℓ_1_‐norm decomposition.

In the case of the numerical phantom data, the dictionary was trained using the fully sampled (ground truth) data. In the case of the ex vivo data, dictionary training was performed for each of the five ex vivo heart samples in turn, using fully sampled data from the remaining four samples. The training data consisted of all voxels containing cardiac tissue, and 1% of the remaining voxels (randomly sampled from air, buffer, and gel). The data were whitened before training. The training was performed using orthogonal matching pursuit 35, with 200 iterations and a batch size of 512. These parameters, provided they were sufficiently large, were not found to significantly affect the dictionaries.

The reconstruction was performed in 3D using MATLAB R2013a (Mathworks, Natick, MA). For the ex vivo data, this required approximately 11 h per reconstruction on a 12 core 2.7 GHz Mac Pro, primarily due to the large number of 3D Fourier transforms associated with the T_2_‐weighting correction, as well as the sparse decomposition step.

### Numerical Phantom Simulation

**Figure 3:**
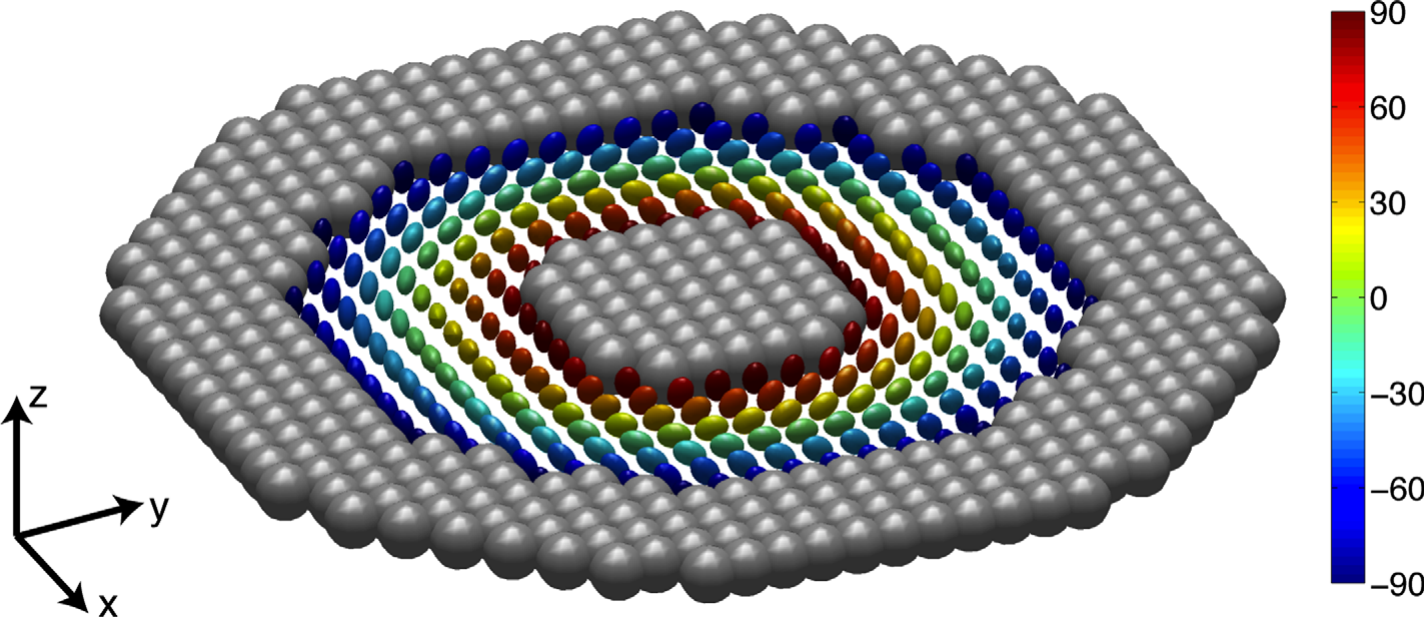
The numerical phantom consisting of concentric annuluses simulating air, gel (outer annulus), tissue (middle annulus) and buffer (inner disc). The gel and buffer components exhibit isotropic diffusion, while the tissue component exhibits orthotropic diffusion. The color of each ellipsoid in the tissue component denotes the helix angle. In the myocardium, the projection of the primary eigenvectors in the short‐axis plane are oriented circumferentially, while the helix angles have a linear transition between the subepicardium and the subendocardium. The tertiary eigenvectors in the myocardium are oriented radially. For display purposes, the phantom has been down‐sampled by a factor of five.

A 2D numerical cardiac phantom, displayed in Figure 3, was generated to evaluate the performance of the CS reconstruction. The geometry and MRI parameters of the phantom simulated a simplified left ventricle. The phantom was oriented in a short‐axis view with the read‐out oriented through‐plane, and the two phase encoding directions oriented in‐plane with an acquisition matrix of 160 × 160. The phantom contained three concentric annuluses corresponding to buffer, tissue, and gel. The T_2_ values of the three components were 40, 24, and 30 ms, respectively. The diffusion in the buffer and gel components was isotropic, with diffusion coefficients of 2.3 and $2.2\times 10^{-3}\;{\mathrm{mm}}^{2}/\text{s}$, respectively.

The signal in the tissue component was generated based on a diffusion tensor model, whose primary, secondary, and tertiary eigenvalues were 1.3, 1.0, and $0.7\times 10^{-3}\;{\mathrm{mm}}^{2}/\text{s}$. Thus, the mean apparent diffusion coefficient (ADC) was $1.0\times 10^{-3}\;{\mathrm{mm}}^{2}/\text{s}$, and the fractional anisotropy (FA) was 0.29. The projection of the primary eigenvectors (**ν**_1_) in the short‐axis plane had a circumferential orientation, and the helix angle (HA), as defined as the elevation angle of **ν**_1_ with respect to the short‐axis plane, had a linear transition between −90° at the outer radius (subepicardium) to 90° at the inner radius (subendocardium). The tertiary eigenvectors (**ν**_3_) were and oriented radially. The secondary eigenvectors (**ν**_2_) were oriented perpendicular to **ν_1_** and **ν**_3_. The proton density of tissue was 80% of that of the buffer and gel. Rician noise was added such that the SNR, defined as the mean divided by the standard deviation in the tissue component of the non‐DW magnitude images, was 60.

### Experimental Validation of Numerical Phantom Simulation

Simulated k‐space data were generated for the phantom in 2D, omitting the read‐out direction. T_2_ decay was simulated according to Eq. [2] for both the retrospective and prospective sampling schemes (i.e., with both constant and variable steps in k_y_). Next, white noise was added independently to the real and imaginary components of k‐space. Finally, undersampling at acceleration factors between two and six was performed. The images were reconstructed as described above. The ground truth consisted of the phantom without noise or T_2_ effects.

The reconstructed magnitude images were fitted with a diffusion tensor model. Parametric maps of mean ADC, FA, and HA were computed. The mean and standard deviation of the mean ADC and FA, and the root‐mean‐squared‐error (RMSE) values of the HA difference maps were calculated. These calculations were performed for voxels corresponding to tissue only.

### Animal Preparation

Hearts were excised from five Sprague‐Dawley rats, weighing between 199 and 221 g, during terminal anaesthesia. Isolated hearts were swiftly perfused in Langendorff constant pressure mode at 80 mmHg with oxygenated (95% O2/5% CO2) Krebs‐Henseleit buffer at 37°C (mM): NaCl 118, KCl 4.7, MgSO4.7H2O 1.2, NaHCO3 25, KH2PO4 1.2, Glucose 11, CaCl2.H2O 1.8, and arrested using high potassium cardioplegic solution. They were subsequently perfusion‐fixed and immersed in isosmotic Karnovsky's fixative with 2 mM Gadolinium contrast agent (Prohance; Bracco, MN). Experimental investigations conformed to the UK Home Office guidance on the Operations of Animals (Scientific Procedures) Act 1986 and were approved by the University of Oxford's ethical review board.

### Experimental Validation of Ex Vivo Data

**Figure 4:**
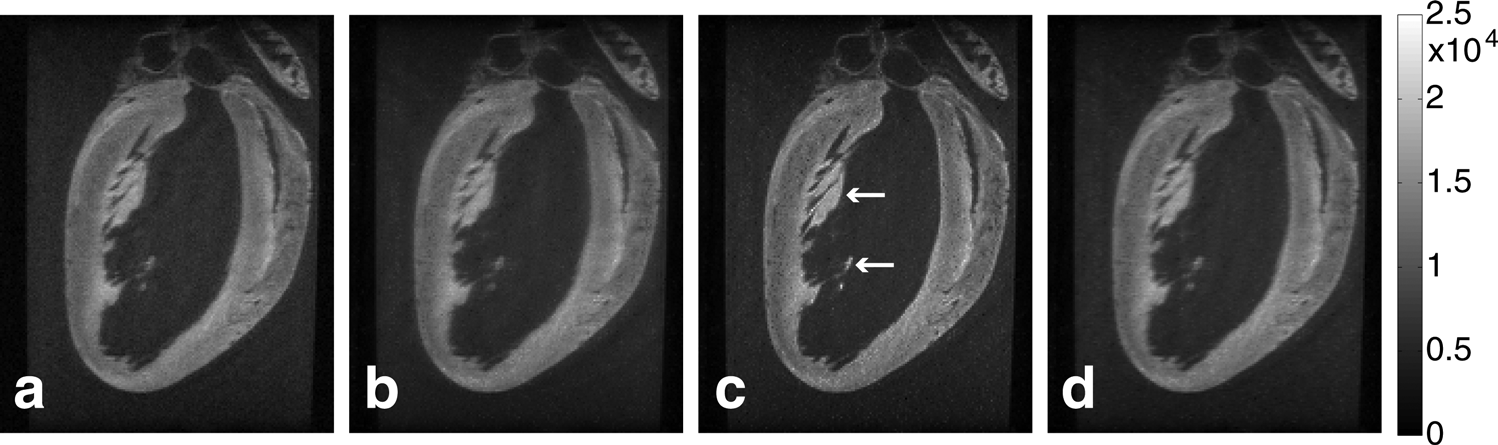
The effect of T_2_‐weighting correction. **a**: Fully sampled ground truth image ( b≠0). **b**: The prospectively 2×‐undersampled and reconstructed image without T_2_‐weighting correction. **c**: The image reconstructed with T_2_‐weighting correction. Arrows indicate regions that have been sharpened by the T_2_‐weighting correction. **d**: The image with T_2_‐weighting matched to the ground truth.

The ex vivo data ground truth consisted of the fully sampled DTI data with 61 diffusion‐encoding directions and 8 b = 0 images. The retrospectively undersampled data consisted of the first 30 diffusion directions and 4 b = 0 images. The sampling locations for the prospectively and retrospectively undersampled data were identical. However, the associated T_2_‐weighting were different in the two schemes. Given that the image reconstruction algorithm incorporates a correction for T_2_‐weighting during the echo train, the reconstructed prospectively undersampled data have a different profile in $k_{y}$ compared with the ground truth. Therefore, for comparison purposes, we applied the T_2_‐weighting profile of the ground truth images to the reconstructed images following reconstruction, so that their T_2_‐weighting profiles were matched. This is illustrated in Figure 4.

The magnitude images for the ground truth and undersampled data were fitted to a diffusion tensor model. The mean and standard deviation of the mean ADC and FA over all myocardial voxels for each sample is reported. The mean and standard deviation RMSE for three parameters (mean ADC, FA, and HA) in the tissue voxels over the five samples is also reported. Wild bootstrapping 36 was used to measure the 95% cone‐of‐uncertainty (COU) of the three eigenvectors in the reconstructed data. The bootstrapping was performed using the Rademacher distribution formulation with 1000 repetitions 37.

Regional analysis of the reconstructed parameter maps was performed in small regions‐of‐interest (ROIs) in the left and right ventricle of a single basal, midventricular, and apical slice (i.e. six ROIs per sample). ROIs were drawn manually in 2D on fully sampled non‐DW images. The right ventricle (RV) ROI enclosed as much of the RV as possible. The left ventricle (LV) ROI enclosed the lateral wall. The RMSE within each ROI was computed for mean ADC, FA, and HA parameter maps. The 95% COU of **ν_1_**, **ν_2_**, and **ν_3_** were also computed in the ROIs.

Fiber tracking was performed along **ν_1_**, **ν_2_**, and **ν_3_** using Diffusion Toolkit and Trackvis 38, in the following data: ground truth, zero‐filled retrospectively 6×  undersampled data, and CS‐reconstructed retrospectively and prospectively 6× undersampled data.

## RESULTS

Table 1 presents the mean of the mean ADC and FA, and the RMSE of HA, in the simulated phantom for both sampling schemes. The mean ADC was overestimated by 0.8–1.1% in the retrospective scheme, and by 1.2–2.2% in the prospective scheme. The FA was underestimated by 3.2–3.9% in the retrospective scheme, and by 4.5–7.3% in the prospective scheme. The RMSE of the HA was 4.33–4.73° in the retrospective scheme, and 5.09–5.73° in the prospective scheme.

**Table 1:** Error Metrics for the Numerical Phantom Reconstructions^a^

Sampling scheme		Acceleration factor				
	Parameter	2	3	4	5	6
Retrospective	Mean apparent diffusion coefficient ( $10^{-3}\;{\mathrm{mm}}^{2}/\text{s}$)	1.01±0.04	1.01±0.05	1.01±0.05	1.01±0.05	1.01±0.05
	Fractional anisotropy	0.28±0.02	0.28±0.02	0.28±0.03	0.28±0.03	0.28±0.03
	Helix angle RMSE (°)	4.33	4.34	4.52	4.63	4.73
Prospective	Mean apparent diffusion coefficient ( $10^{-3}\;{\mathrm{mm}}^{2}/\text{s}$)	1.01±0.05	1.02±0.06	1.02±0.06	1.02±0.07	1.02±0.07
	Fractional anisotropy	0.28±0.02	0.27±0.02	0.27±0.03	0.27±0.03	0.27±0.03
	Helix angle RMSE (°)	5.09	5.65	5.66	5.72	5.73

a In the case of the mean apparent diffusion coefficient and fractional anisotropy, the mean and standard deviation over all voxels corresponding to tissue is presented. In the case of the helix angle, the RMSE is presented.

**Figure 5:**
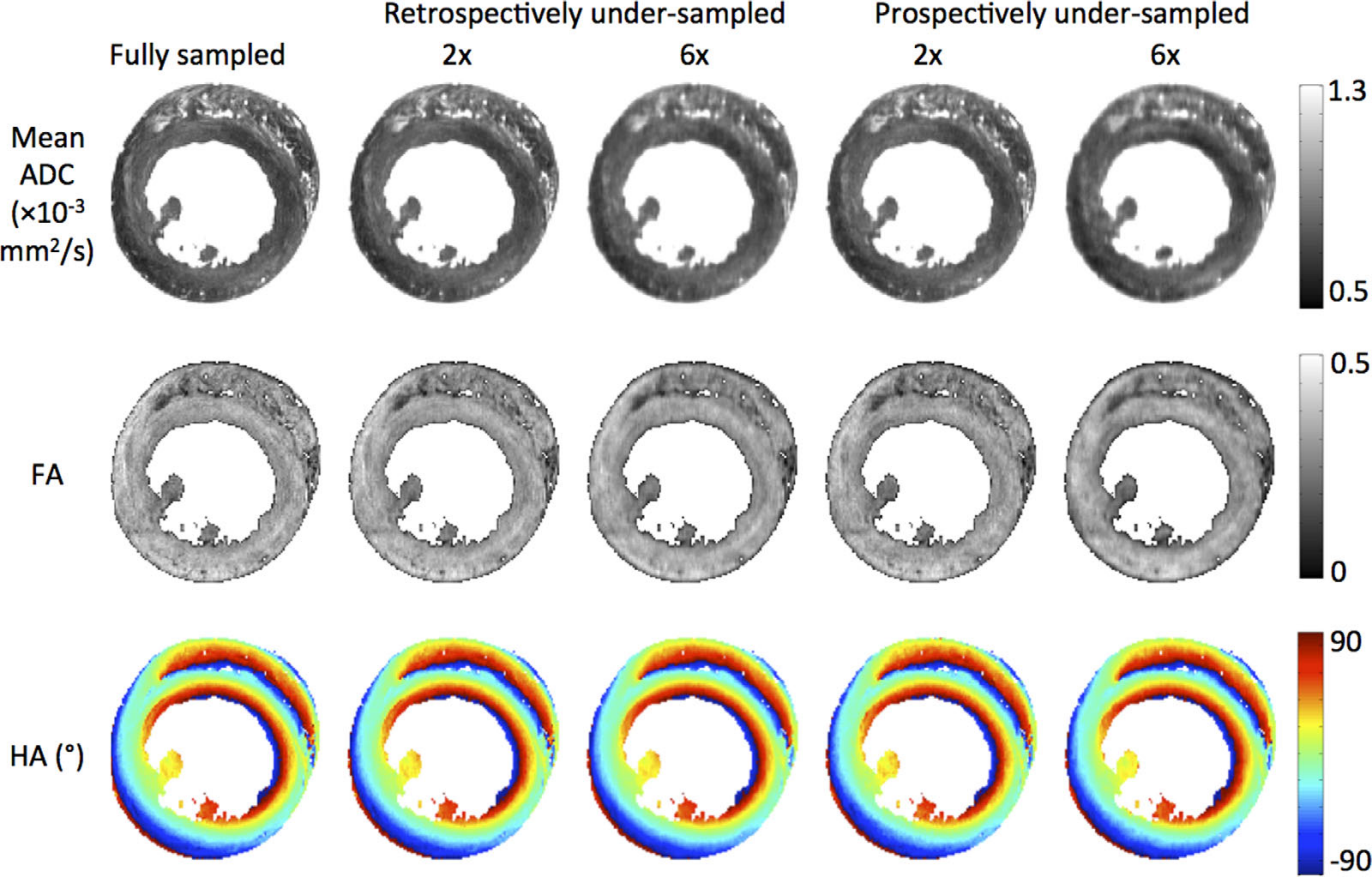
Parameter maps of mean apparent diffusion coefficient (ADC), fractional anisotropy (FA) and helix angle (HA) at acceleration factors of 2 and 6 for retrospectively and prospectively undersampled data with matched T_2_‐weighting.

Figure 5 displays T_2_‐matched mean ADC, FA, and HA maps in a single sample based on retrospective and prospective undersampling at acceleration factors of two and six. In general, the retrospectively undersampled reconstructed images were visually sharper than the prospectively undersampled reconstructed images. The images became increasingly unsharpened with increased acceleration.

As per the phantom experiments, FA was underestimated as a result of the prospective undersampling and reconstruction. Table 2 presents the mean and standard deviation of the mean ADC and FA over all voxels in the myocardium for prospectively undersampled data. At an acceleration factor of two, the mean ADC was approximately 0.5% higher, and the FA was approximately 2.5% lower, than the ground truth. At an acceleration factor of six, the mean ADC was approximately 1% lower, and the mean FA was approximately 5% lower, than the ground truth. The standard deviation of both parameters also decreased with increased acceleration.

**Table 2:** Mean and RMSE of Mean ADC, FA, HA, and 95% Cones of Uncertainty for Acceleration Factors between Two and Six for Prospectively Undersampled T_2_w‐Matched Data^a^

Parameter	Ground truth	Acceleration factor				
		2	3	4	5	6
Mean ADC ( ${\times 10}^{-3}\;{\mathrm{mm}}^{2}/\text{s}$)	1.07±0.02	1.07±0.02	1.07±0.02	1.06±0.02	1.07±0.02	1.07±0.02
Mean FA	0.26±0.01	0.26±0.01	0.25±0.01	0.25±0.01	0.25±0.01	0.25±0.01
ADC RMSE $\left(\times 10^{-5}\;{\mathrm{mm}}^{2}/\text{s}\right)$	‐	3.78±0.17	4.84±0.17	5.57±0.17	6.14±0.17	6.64±0.16
FA RMSE $\left(\times 10^{-2}\right)$	‐	2.24±0.06	2.85±0.19	2.83±0.12	3.00±0.11	3.16±0.13
HA RMSE (°)	‐	4.96±0.44	5.70±0.43	5.99±0.50	6.40±0.64	6.61±0.66
**v_1_** COU (°)	3.7±0.2	4.7±0.2	2.9±0.3	2.9±0.2	3.0±0.2	3.0±0.3
**v_2_** COU (°)	14.7±1.0	19.4±1.4	12.1±1.4	12.1±1.3	12.3±1.3	12.3±1.4
**v_3_** COU (°)	14.4±1.0	19.4±1.5	11.8±1.5	11.8±1.4	12.1±1.4	12.0±1.4

Mean and standard deviation are reported over five hearts.

**Figure 6:**
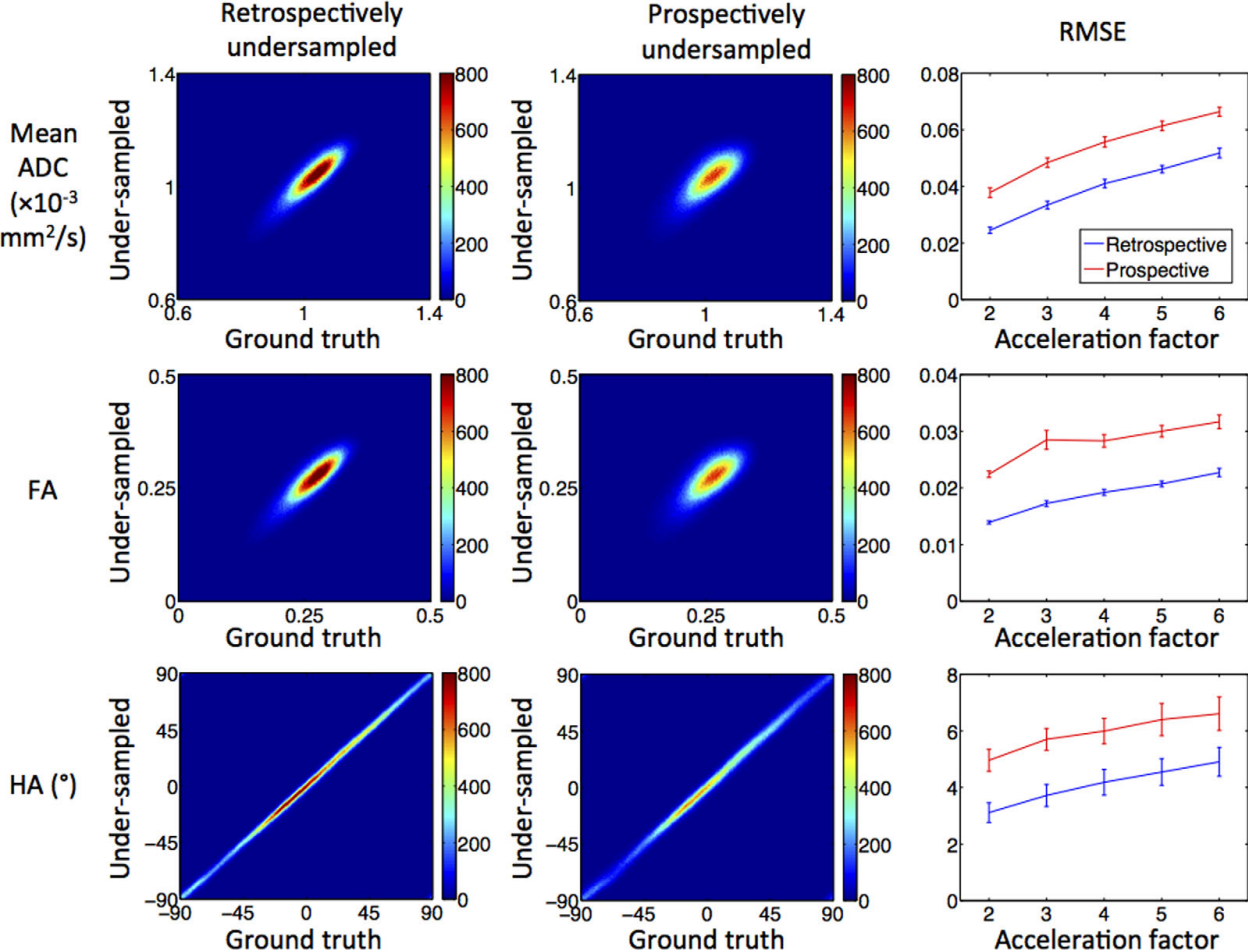
Joint histograms of mean apparent diffusion coefficient (ADC), fractional anisotropy (FA) and helix angle (HA) for 6× accelerated retrospectively (left column) and prospectively (middle column) undersampled data in the global myocardium. Right column: Root mean‐squared error (RMSE) versus acceleration factor for mean ADC, FA, and HA. The mean and standard deviation of the RMSE over the five samples is presented.

In the retrospectively undersampled data, the mean RMSE of the mean ADC over the five samples was $2.4\;\times \;10^{-5}\;{\mathrm{mm}}^{2}/\text{s}$ for an acceleration factor of two, increasing to $5.2\;\times \;10^{-5}\;{\mathrm{mm}}^{2}/\text{s}$ at an acceleration factor of six. The mean RMSE of the FA was 0.014 for an acceleration factor of two, and 0.023 for an acceleration factor of six. The mean RMSE of the HA was 3.1° for an acceleration factor of two, and 4.9° for an acceleration factor of six. Joint histograms of mean ADC, FA, and HA at 6× acceleration are shown in Figure 6. The spread of the data is larger for all parameters in prospectively undersampled data, and increases with acceleration. Figure 6 also displays the RMSE values for mean ADC, FA, and HA. These data are also presented in Table 2 for the prospectively undersampled data, as is the COU values for the three eigenvectors.

The RMSE values of the prospectively undersampled data were higher than those of the retrospectively undersampled data. The mean RMSE of the mean ADC was $3.8\;\times \;10^{-5}\;{\mathrm{mm}}^{2}/\text{s}$ and $6.7\;\times \;10^{-5}\;{\mathrm{mm}}^{2}/\text{s}$ for acceleration factors of two and six, respectively. The mean RMSE of the FA was 0.022 for an acceleration factor of two, and 0.032 for an acceleration factor of six. Finally, the mean RMSE of the HA was 5.0° for an acceleration factor of two, and 6.6° for an acceleration factor of six.

Although the RMSE for mean ADC, FA, and HA increases with acceleration factor, the cones of uncertainty for all three eigenvectors decreased for acceleration factors of three or greater. Wild bootstrapping yielded 95% COU in **v**_1_ of 4.7° and 3.0° at acceleration factors of two and six, respectively, in the prospectively sampled data, and 3.7° in the ground truth data. The COU in **v**_2_ was 19.4° and 12.3° at factors of two and six, respectively, and 14.7° in the ground truth data. The COU in **v**_3_ was 19.0° and 12.0° at factors of two and six, and 14.4° in the ground truth data.

Regional analysis showed that the RMSE in the LV was lower than the RV. At an acceleration factor of two and in a midventricular slice, the mean ADC RMSE in the LV was 31% lower, the FA RMSE was 17% lower, and the HA was 47% lower than the RV. At an acceleration factor of six, the mean ADC RMSE in the LV was 36% lower, the FA RMSE was 31% lower, and the HA RMSE was 59% lower than in the RV. In the LV, the mean ADC, FA, and HA RMSE values were lowest in the midventricular slice. In the RV, the RMSE was lowest in the apical slice for the ADC, basal slice for the FA, and midventricular slice for the HA. The 95% COU of **v**_1_ was lowest in the midventricular slice for both the LV and RV. The 95% COU of **v**_1_ was consistently lower in the LV than in the RV. In the case of **v**_2_ and **v**_3_, the 95% COU in the LV was higher in the basal slice, approximately equal in the midventricular slice, and lower in the apical slice than in the RV.

**Figure 7:**
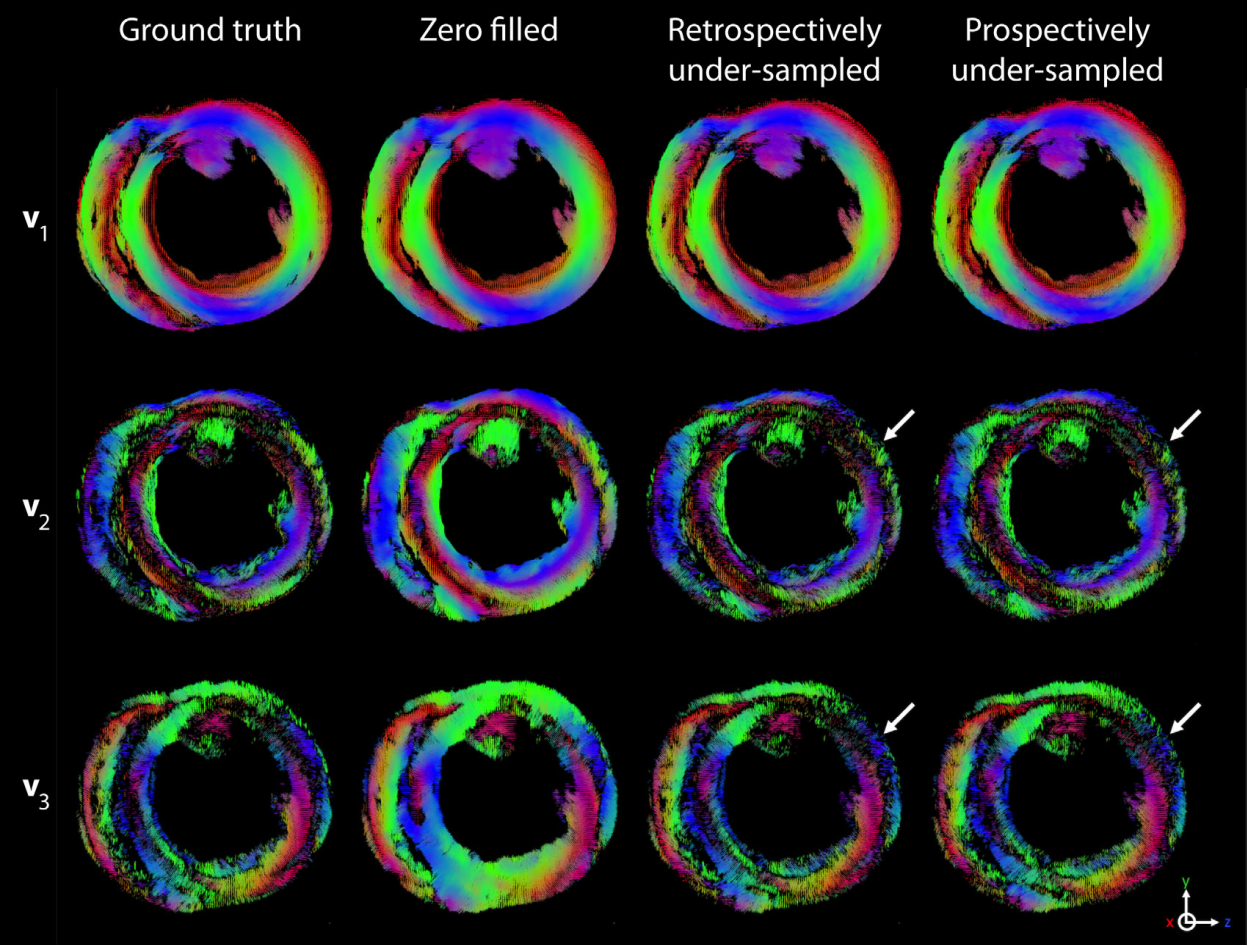
Fiber tracking in a 5‐voxel‐thick mid‐myocardial axial slab based on the 1^st^, 2^nd^, and 3^rd^ eigenvectors (**ν**_1_, **ν**_2_, and **ν**_3_; top, middle, bottom). Tracts were reconstructed from ground truth data, zero‐filled retrospectively undersampled data, CS‐reconstructed retrospectively undersampled data, and CS‐reconstructed prospectively undersampled data (left to right). In the undersampled images, the acceleration factor was 6. Tracts based on **ν**_1_ were virtually indistinguishable in each case. While significant smoothing of **ν**_2_ and **ν**_3_ tracts were observed in the tracts reconstructed using the zero‐filled data, these tracts were well preserved in the CS‐reconstructed data when compared with the ground truth. Marginal differences observed in the lateral wall are highlighted with arrows.

Figure 7 illustrates fiber tracking in the whole heart based on **ν**_1_, **ν**_2_, and **ν_3_**. Fiber tracts based on **ν**_1_ in each undersampled case were virtually indistinguishable from the ground truth data. Smoothing of **ν**_2_ and **ν**_3_ tracts are prominent in the zero‐filled retrospectively undersampled data. In contrast, **ν**_2_ and **ν**_3_ tracts were well preserved in the CS‐reconstructed cases, with marginal differences in the lateral wall of the midaxial myocardium.

## DISCUSSION

In this work, a novel dictionary‐based approach is proposed and evaluated for accelerating DTI. Prospective undersampling up to a factor of 6 × was performed, reducing the total acquisition time from 7 h 30 min to 1 h 15 min. Furthermore, the correction of artifacts arising from discontinuous T_2_‐weighting is incorporated into the reconstruction algorithm.

We observed that the prospectively undersampled data had higher errors than the retrospectively undersampled data. Our simulations show that this arises from the difference in T_2_‐weighting between the two schemes. As a result of the narrower grouping of the initial echoes about the center of $k_{y}$ in the prospectively undersampled data, its SNR is lower than that of the retrospectively undersampled data. The corollary is that for echo train imaging sequences, additional shots and time would be required to provide the same T_2_‐weighting as the retrospectively undersampled data. Therefore, it is more accurate to state that the SNR in retrospectively undersampled data is artificially enhanced. While for single‐echo imaging sequences such as a spin or stimulated echo sequences variable T_2_‐weighting is not an issue, these sequences require significantly longer acquisition times (e.g., factor of 8 in this case).

The variable band spacing in our prospective undersampling scheme increasingly unsharpened the point spread function as the acceleration factor increased. The T_2_‐weighting correction compensates for this and amplifies the edges of k‐space. This leads to sharper but noisier images and truncation artefacts. However, these artefacts are mitigated by the total variation (image smoothness) component of the reconstruction algorithm.

Acquiring a T_2_ map added 1 h 46 min to the total scan time. It has been shown that discontinuous T_2_‐weighting in fast spin echo can be corrected using only a single excitation, adding just seconds to the scan time 31. However, this approach assumes that the data in each echo train can be characterized by a single T_2_, and is better suited for homogeneous samples. Alternatively, acquiring a lower resolution T_2_ map should suffice, thus reducing the scan time overhead. In the absence of a T_2_ map, the T_2_‐weighting correction can be omitted from the reconstruction algorithm, yielding images with the same T_2_‐weighting as the acquired data. However, in this work it was necessary to correct the T_2_‐weighting to compare the reconstructed images with the ground truth.

Comparing the RMSE values for the retrospectively undersampled reconstructions with those from a recent distributed compressed sensing study using the wavelet transform 10, we found that our mean RMSE values were 60% lower for FA and 30% lower for HA. The mean RMSE values for mean ADC were comparable in both studies. We note, however, that the mean ADC in the samples of 10 were considerably lower (< 50%) than the mean ADC reported in this study, and thus the mean RMSE as a proportion of the mean ADC was also lower using our approach. The differences in mean ADC may have stemmed from differences in sample preparation, such as the fixative used and the ambient temperature.

Regarding the mean values of tensor parameters over the myocardium, the mean ADC was relatively robust to acceleration, being underestimated in prospectively 6× undersampled data by approximately 2.5%. The fractional anisotropy were less robust to acceleration, being underestimated by approximately 5%. This bias was also observed in the simulated phantom reconstructions, and is most likely a result of the dictionary not being sufficiently flexible in describing anisotropic diffusion at a range of orientations. Future research will focus on optimal representation of diffusion signals using dictionaries. Furthermore, the system would be more practical without the requirement of external training data. Dictionary training based on undersampled data will also be investigated in future work.

The 95% COU values for the reconstructed data were low, indicating a high level of precision. The COU values for an acceleration factor of two were higher than those of the ground truth data, possibly as a result of the T_2_‐weighting correction amplifying high frequency data. At acceleration factors of three to six, the compressed sensing reconstruction has a de‐noising effect, yielding lower COU values than the ground truth.

Regional analysis showed that the reconstruction performance in the left ventricle was better than in the right ventricle. One reason for this is that the LV is larger than the RV, and less susceptible to partial voluming. The loss of high frequency data will, therefore, have a greater impact on reconstruction of the right ventricle. In addition, the 95% COU in **ν**_1_ is higher in the RV than in the LV. The reconstruction of the LV in the midventricular ROI was better than in the apical or basal ROIs. The midventricular LV ROI also had the lowest 95% COU in **ν**_1_. This could be related to the higher SNR at the center of the RF coil.

The fiber tracking results indicate that tracking of **ν**_1_ is relatively robust to undersampling, even with simple zero‐filling. On the other hand, **ν**_2_ and **ν**_3_ are harder to reliably measure. Here, CS‐reconstruction of both retrospectively and prospectively undersampled data reconstructed with CS show excellent agreement in **ν**_1_, **ν**_2_, and **ν**_3_ tracts compared with the ground truth.

Although the diffusion tensor model was fit in this work, the sampling scheme and reconstruction pipeline is not limited to diffusion tensor imaging. The algorithm reconstructs diffusion‐weighted images without fitting an explicit model of diffusion to the data. Therefore, this algorithm could be used to reconstruct data with a higher number of samples in q‐space, such as in diffusion spectrum imaging.

## CONCLUSIONS

A novel system for accelerating DTI that combines a fast imaging sequence with compressed sensing was implemented. This was achieved using a variable density phase encoding scheme for undersampling 3D FSE data, and a compressed sensing framework incorporating adaptive dictionaries and T_2_‐weighting correction for reconstruction of undersampled DTI data. We acquired DTI data prospectively undersampled in k‐space by up to a factor of 6. This combined with an echo train length of 8 to reduce effective scan times by up to 48 × as compared to fully sampled spin echo imaging. The quality of reconstructions of undersampled data compared favorably to the literature, and the reconstructed parameter maps and fiber tracking were in good agreement with the ground truth data. The combination of echo train sequences and compressed sensing can dramatically reduce acquisition times in DTI, and this could lead to improvements in spatial resolution, flexibility in diffusion schemes, and clinical feasibility.

## References

[1] Holmes AA , Scollan DF , Winslow RL. Direct histological validation of diffusion tensor MRI in formaldehyde‐fixed myocardium. Magn Reson Med 2000;44:157–161. 10893534 10.1002/1522-2594(200007)44:1<157::aid-mrm22>3.0.co;2-f

[2] Kung GL , Nguyen TC , Itoh A , Skare S , Ingels NB Jr , Miller DC , Ennis DB. The presence of two local myocardial sheet populations confirmed by diffusion tensor MRI and histological validation. J Magn Reson Imaging 2011;34:1080–1091. 21932362 10.1002/jmri.22725 PMC3195899

[3] Basser PJ , Mattiello J , LeBihan D. MR diffusion tensor spectroscopy and imaging. Biophys J 1994;66:259–267. 8130344 10.1016/S0006-3495(94)80775-1 PMC1275686

[4] Hsu EW , Henriquez CS. Myocardial fiber orientation mapping using reduced encoding diffusion tensor imaging. J Cardiovasc Magn Reson 2001;3:339–347. 11777226 10.1081/jcmr-100108588

[5] Rapacchi S , Wen H , Viallon M , Grenier D , Kellman P , Croisille P , Pai VM. Low b‐value diffusion‐weighted cardiac magnetic resonance imaging: initial results in humans using an optimal time‐window imaging approach. Invest Radiol 2011;46:751–758. 21691213 10.1097/RLI.0b013e31822438e8 PMC3397777

[6] Lau AZ , Tunnicliffe EM , Frost R , Koopmans PJ , Tyler DJ , Robson MD. Accelerated human cardiac diffusion tensor imaging using simultaneous multislice imaging. Magn Reson Med 2015;73:995–1004. 24659571 10.1002/mrm.25200

[7] Lustig M , Donoho D , Pauly JM. Sparse MRI: the application of compressed sensing for rapid MR imaging. Magn Reson Med 2007;58:1182–1195. 17969013 10.1002/mrm.21391

[8] Ma HT , Zhang LM , Ren R , Wu SH. Compressed sensing on DTI via rotating interpolation. In Proceedings of the TENCON 2013 ‐ 2013 IEEE International Conference of IEEE Region 10, Xi'an, China 2013.

[9] Paquette M , Merlet S , Gilbert G , Deriche R , Descoteaux M. Comparison of sampling strategies and sparsifying transforms to improve compressed sensing diffusion spectrum imaging. Magn Reson Med 2015;73:401–416. 24478106 10.1002/mrm.25093

[10] Wu Y , Zhu YJ , Tang QY , Zou C , Liu W , Dai RB , Liu X , Wu EX , Ying L , Liang D. Accelerated MR diffusion tensor imaging using distributed compressed sensing. Magn Reson Med 2014;71:763–772. 23494999 10.1002/mrm.24721.

[11] Michailovich O , Rathi Y , Dolui S. Spatially regularized compressed sensing for high angular resolution diffusion imaging. IEEE Trans Med Imaging 2011;30:1100–1115. 21536524 10.1109/TMI.2011.2142189 PMC3708319

[12] Mani M , Jacob M , Guidon A , Magnotta V , Zhong J. Acceleration of high angular and spatial resolution diffusion imaging using compressed sensing with multichannel spiral data. Magn Reson Med 2015;73:126–138. 24443248 10.1002/mrm.25119

[13] Landman BA , Bogovic JA , Wan H , El Zahraa ElShahaby F , Bazin PL , Prince JL. Resolution of crossing fibers with constrained compressed sensing using diffusion tensor MRI. Neuroimage 2012;59:2175–2186. 22019877 10.1016/j.neuroimage.2011.10.011 PMC3254826

[14] Hales PW , Schneider JE , Burton RA , Wright BJ , Bollensdorff C , Kohl P. Histo‐anatomical structure of the living isolated rat heart in two contraction states assessed by diffusion tensor MRI. Prog Biophys Mol Biol 2012;110:319–330. 23043978 10.1016/j.pbiomolbio.2012.07.014 PMC3526796

[15] Bilgic B , Setsompop K , Cohen‐Adad J , Yendiki A , Wald LL , Adalsteinsson E. Accelerated diffusion spectrum imaging with compressed sensing using adaptive dictionaries. Magn Reson Med 2012;68:1747–1754. 23008145 10.1002/mrm.24505 PMC3504650

[16] Gramfort A , Poupon C , Descoteaux M. Denoising and fast diffusion imaging with physically constrained sparse dictionary learning. Med Image Anal 2014;18:36–49. 24084469 10.1016/j.media.2013.08.006

[17] Adluru G , Hsu E , Di Bella EVR. Constrained reconstruction of sparse cardiac MR DTI data. Functional imaging and modeling of the heart. New York: Springer; 2007 p 91–99.

[18] Welsh CL , Dibella EV , Adluru G , Hsu EW. Model‐based reconstruction of undersampled diffusion tensor k‐space data. Magn Reson Med 2013;70:429–440. 23023738 10.1002/mrm.24486 PMC4469271

[19] Gao H , Li L , Zhang K , Zhou W , Hu X. PCLR: phase‐constrained low‐rank model for compressive diffusion‐weighted MRI. Magn Reson Med 2014;72:1330–1341. 24327553 10.1002/mrm.25052 PMC4372802

[20] Menzel MI , Tan ET , Khare K , Sperl JI , King KF , Tao X , Hardy CJ , Marinelli L. Accelerated diffusion spectrum imaging in the human brain using compressed sensing. Magn Reson Med 2011;66:1226–1233. 22012686 10.1002/mrm.23064

[21] Aboussouan E , Marinelli L , Tan E. Non‐Cartesian compressed sensing for diffusion spectrum imaging. In Proceedings of the 19th Annual Meeting of ISMRM, Montreal, Quebec, Canada, 2011. Abstract 1919.

[22] Saint‐Amant E , Descoteaux M. Sparsity characterisation of the diffusion propagator. In Proceedings of the 19th Annual Meeting of ISMRM, Montreal, Quebec, Canada, 2011. Abstract 1915.

[23] Merlet SL , Deriche R. Continuous diffusion signal, EAP and ODF estimation via compressive sensing in diffusion MRI. Med Image Anal 2013;17:556–572. 23602920 10.1016/j.media.2013.02.010

[24] Aharon M , Elad M , Bruckstein A. K‐ SVD: an algorithm for designing overcomplete dictionaries for sparse representation. IEEE Trans Signal Process 2006;54:4311–4322.

[25] Mairal J , Bach F , Ponce J , Sapiro G. Online learning for matrix factorization and sparse coding. J Mach Learn Res 2010;11:19–60.

[26] McClymont D , Teh I , Whittington H , Schneider J. Compressed sensing reconstruction of prospectively under‐sampled cardiac diffusion tensor MRI. In Proceedings of the 23rd Annual Meeting of ISMRM, Toronto, Canada, 2015. Abstract 3695.

[27] Caruyer E , Lenglet C , Sapiro G , Deriche R. Design of multishell sampling schemes with uniform coverage in diffusion MRI. Magn Reson Med 2013;69:1534–1540. 23625329 10.1002/mrm.24736 PMC5381389

[28] Awate SP , DiBella EVR. Compressed sensing hardi via rotation‐invariant concise dictionaries, flexible k‐space undersampling, and multiscale spatial regularity. In Proceedings of the IEEE 10th International Symposium on Biomedical Imaging (ISBI), San Francisco, California, USA, 2013 p 9–12.

[29] Gudbjartsson H , Patz S. The rician distribution of noisy MRI data. Magn Reson Med 1995;34:910–914. 8598820 10.1002/mrm.1910340618 PMC2254141

[30] Pandit P , Qi Y , King KF , Johnson GA. Reduction of artifacts in T2‐weighted PROPELLER in high‐field preclinical imaging. Magn Reson Med 2011;65:538–543. 20928875 10.1002/mrm.22624 PMC3026877

[31] Zhou X , Liang Z‐P , Cofer GP , Beaulieu CF , Suddarth SA , Johnson GA. Reduction of ringing and blurring artifacts in fast spin‐echo imaging. J Magn Reson Imaging 1993;3:803–807. 8400569 10.1002/jmri.1880030518

[32] Candès EJ , Wakin MB , Boyd SP. Enhancing sparsity by reweighted ℓ 1 minimization. J Fourier Anal Appl 2008;14:877–905.

[33] Daducci A , Van De Ville D , Thiran JP , Wiaux Y. Sparse regularization for fiber ODF reconstruction: from the suboptimality of l2 and l1 priors to l0. Med Image Anal 2014;18:820–833. 24593935 10.1016/j.media.2014.01.011

[34] Tibshirani R. Regression shrinkage and selection via the lasso. J R Stat Soc Series B Stat Methodol 1996:267–288.

[35] Tropp JA , Gilbert AC. Signal recovery from random measurements via orthogonal matching pursuit. IEEE Trans Inf Theory 2007;53:4655–4666.

[36] Whitcher B , Tuch DS , Wisco JJ , Sorensen AG , Wang L. Using the wild bootstrap to quantify uncertainty in diffusion tensor imaging. Hum Brain Mapp 2008;29:346–362. 17455199 10.1002/hbm.20395 PMC6870960

[37] Davidson R , Flachaire E. The wild bootstrap, tamed at last. J Econom 2008;146:162–169.

[38] Wang R , Benner T , Sorensen AG , Wedeen VJ. Diffusion toolkit: a software package for diffusion imaging data processing and tractography. In Proceedings of the Joint Annual Meeting of ISMRM‐ESMRMB, Berlin, Germany, 2007.

